# Type 1C Multiple Symmetrical Lipomatosis: A Cause of Misdiagnosis in Females

**DOI:** 10.7759/cureus.40970

**Published:** 2023-06-26

**Authors:** Abdullah Şükün, Mehmet Fatih Demirci, Ertan Akbay

**Affiliations:** 1 Department of Radiology, Başkent University Alanya Research and Application Center, Antalya, TUR; 2 Department of Internal Medicine, Başkent University Alanya Research and Application Center, Antalya, TUR; 3 Department of Cardiology, Başkent University Alanya Research and Application Center, Antalya, TUR

**Keywords:** fat accumulation, lipom, madelung’s disease, benign symmetric lipomatosis, multiple symmetrical lipomatosis (msl)

## Abstract

Multiple symmetrical lipomatosis (MSL) is a disease that causes symmetrical fat deposits in the neck, shoulders, and upper trunk. It is more common in the neck area in men who consume alcohol. The male-to-female ratio varies from 15:1 to 30:1. Madelung’s disease has been reported in a small number of female patients who do not consume alcohol. Pseudoathletic appearance (MSL type 1C) is rare and causes misdiagnosis. We would like to present a 50-year-old woman with an athletic appearance who had fat deposits on her shoulders and upper chest. After excluding obesity and Cushing's syndrome, which were initially considered, we aimed to remind people of this entity that causes symmetrical fat deposits in the upper trunk in females.

## Introduction

Benjamin Brodie initially described Madelung's illness, also known as multiple symmetrical lipomatosis (MSL) or benign symmetric lipomatosis, as an uncommon pathology of adipose metabolism in 1846. In the craniofacial region, neck, shoulder, trunk, limbs, and other places, it manifests as many symmetrical, nonencapsulated fatty lumps [1]. The most common symptom of neck involvement is a distinct indication termed a Madlung's or horse collar. Middle-aged males of Mediterranean descent are more likely to have MSL, which is linked to alcohol usage [2]. MSL has been reported in a small number of female patients who do not consume alcohol. Chronic excessive alcohol use has been associated with the progression of lipomatosis in some MSL patients. Large-scale deletions and specific point mutations in mitochondrial DNA (mtDNA) have been reported in a small proportion of patients [3,4].

MSL disease is more easily recognized by alcohol use and symmetrical fat accumulation in the neck in men. We would like to present a 50-year-old woman with an athletic appearance who had fat deposits on her shoulders and upper chest. After excluding obesity and Cushing's syndrome, which were initially considered, we aimed to remind people of this entity that causes symmetrical fat deposits in the upper trunk in females.

## Case presentation

A 50-year-old female presented with complaints of swelling in the shoulders, back, and chest and deep breathing. She was examined at an external center, told that there was a blockage in the lymph vessels, and referred to us for heart disease. Chest pain was not detected, but she was breathing deeply.

**Figure 1:**
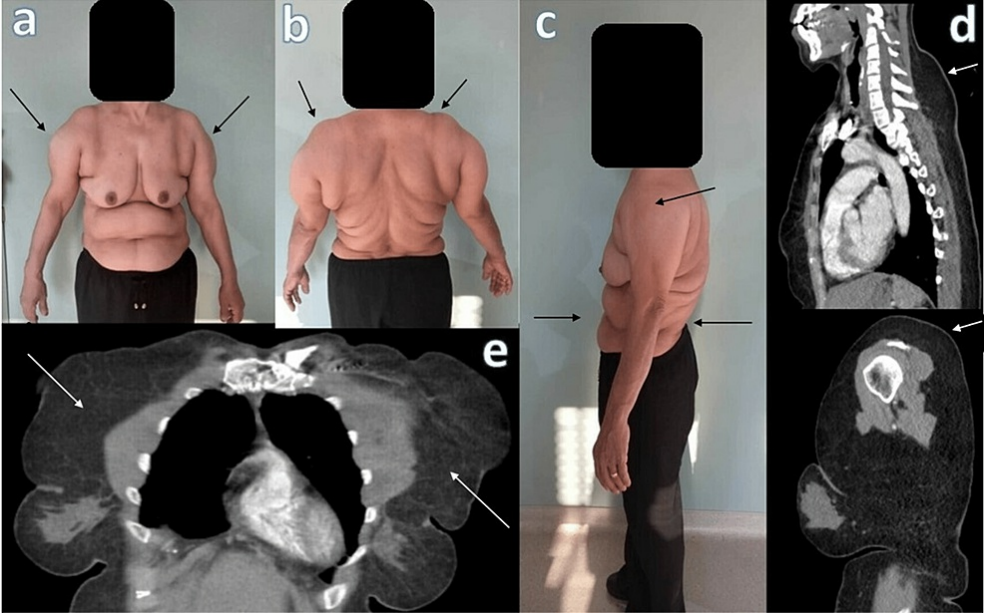
Clinical and radiologic images of the case (a,b) In the anterior and posterior view of the patient, a pseudoathletic appearance is prominent in the shoulder region (arrows). (c) Sagittal view shows prominent appearance in the deltoid muscle region and diffuse pseudoathletic appearance in the anterior and posterior upper body (arrows). (d) Sagittal view shows adipose tissue hypertrophy in the neck region on sagittal tomography image. Sagittal upper extremity image shows non-capsulated adipose tissue around the deltoid muscle (arrows). (e) Coronal image of the upper extremity shows marked hypertrophy of adipose tissue around the pectoral muscles (arrows).

The patient had no known medical disease. A cardiovascular system examination revealed a blood pressure of 150/90 mm Hg. The heart was rhythmic, and no murmur was detected. The ejection fraction was found to be 65% on echo. The intraarterial septum was thin. A suspected left-to-right shunt was observed. Transesophageal echocardiography was recommended. Transesophageal echocardiography revealed no shunt. The aortic valve degenerated. All laboratory values were normal. On physical examination, the patient had a muscular appearance in the upper extremities, shoulders, and trunk (Figure 1).

Initially, a bilateral venous Doppler examination was ordered to evaluate these structures for arteriovenous evaluation and tissue characteristics. Vascular structures were normal on Doppler ultrasonography. Superficial ultrasonography showed a symmetric non-capsulated adipose tissue increase. No encapsulated lipoma with visible borders was detected. All findings were normal, suggesting multiple symmetric lipomatoses (Madelung's disease).

An internal medicine doctor consulted with the patient after ruling out other diagnoses. It was evident from the internal medicine physician's evaluation that the patient had received cortisone from the endocrine physician five years prior due to muscle thickening in the arms but had not used it. Cortisol, growth hormone, IGF-1, cortisol, and TSH were evaluated in the laboratory examinations of the patient, and all values were normal (Table 1). A thoracic computed tomography (CT) scan showed normal lung parenchyma and suprarenal glands. The patient was not taking any medication other than vitamin D. The patient had no history of alcohol abuse. Prominent bilateral symmetric lipomas were found on the shoulders, and MSL was diagnosed (Figure 1). Family history revealed a similar phenotype in five siblings.

**Table 1:** Laboratory findings of the patient

		Unit	Reference range
Growth hormone	0.014	µg/L	0.01–3.60
IGF-1	57.2	ng/mL	50–252
Cortisol	12	µg/dL	3.7–19.4
TSH	4.62	U/L	0.35–4.94
T4	0.82	ng/dL	0.61–1.12
Folate	13.15	ng/mL	3.1–19.9
Vitamin B12	363	pg/mL	211–911
Vitamin D	7.36	ng/mL	10,6–43,4
Ferritin	4.6	ng/mL	10–291
Glucose	82	mg/dL	74–106
Glucose (postprandial 2 hours)	110	mg/dL	70–140
Bilirubin (total)	0.95	mg/dL	0.2–1.2
Iron (serum)	63	µg/dL	40–170
LDL	130	mg/dL	50–150
HDL	56	mg/dL	40–60
Triglyceride	82	mg/dL	0–150
Cholesterol	200	mg/dL	0–200
Creatinine	0.6	mg/dL	0.6–1.1
Calcium	8.8	mg/dL	8.6–10.8
Uric acid	2.5	mg/dL	2.6–6
HGB	12.1	g/dL	12.16
Rheumatoid factor (RF)	4	IU/mL	0–30
CRP	0.8	mg/L	0–5
Anti-nuclear antibody	Negative		

## Discussion

According to epidemiologic studies, the prevalence rate of MSL is 15:1 to 30:1 higher in men than in women. Due to the low incidence rate (1 in 25,000) and late age of onset, the genesis and pathology of the disease are not yet fully understood [5]. The mechanism is thought to include defects in the mitochondrial respiratory chain, deletions, and punctiform mutations on mitochondrial DNA associated with lipolysis and catecholamine-induced lipogenesis alterations. Adipogenesis is thought to be due to an exaggerated hyperplastic proliferation of the subcutaneous brown adipose tissue. The pathogenesis of MSL disease is mostly characterized by the generation of new adipocytes rather than the expansion of existing cells [6]. The MSL comes in three different forms: Neck distribution type 1, pseudo-athletic look type 2, and gynecoid appearance type 3 [7]. The two traditional treatments for MSL are lipectomy and liposuction; however, they are typically merely palliative, and it is unclear if they are effective. Interventions are only used to treat symptoms since Madelung's disease's origin and pathophysiology are yet unknown. Nonetheless, the decision to have surgery should be founded on a careful assessment of the disease's severity, the location of fat deposits, patient expectations, and the surgeon's experience [8]. There have been more than 400 cases reported in the literature, although only a few of the individuals appeared to be pseudoathletic. The lower part of the trunk and belly remain unaffected in the typical type of MSL when lipomas develop along the neck, upper trunk, and proximal upper extremities. Because of fatty deposits pushing on the cervical area, patients with MSL may experience functional symptoms such as dysphagia, odynophagia, or hoarseness. The pseudoathletic look of MSL, in particular, is less widely understood and may go untreated for years. A thorough clinical history is crucial [9]. The localized form with symmetrical lipomas in the neck region (buffalo hump), which is more common in men, is associated with alcohol consumption. Liver disease is a common comorbidity of MSL, and acute renal failure may also be seen [10]. In non-alcoholic type 2 MSL, a pseudoathletic appearance may be misinterpreted as obesity. The differential diagnosis of obesity may also include other conditions such as familial multiple lipomatosis, Cushing's syndrome, iatrogenic cutaneous lipomatosis, encapsulated lipomas, angiolipomatosis, myxoid liposarcoma, and lymphoma [9,11]. An essential diagnostic tool for MSL disease is computed tomography with multiplanar reconstruction (MPR) and volumetric rendering technique (VRT), which allows for the evaluation of the severity of lesions and the planning of treatments. Clinical data also allow for the differentiation of MSL disease from other conditions characterized by an excessive accumulation of adipose tissue [12]. Fifty-four people with MSL illnesses were involved in a review study conducted in China. With a frequency of 1.85%, there was just one female patient among all of them. Endocrine system disorders were shown to be the most prevalent comorbidity, accounting for 81.48% of all comorbidities in patients with MSL. It was interesting to discover that 20.37% of cases were complicated by malignancies, particularly malignancies of the digestive tract. The dismal outlook for MSL may be explained by its many comorbidities. A postoperative recurrence occurred in nearly 40% of all patients, who had surgical therapy in more than 70% of cases [13]. In a study investigating postoperative recurrence in MSL patients, a combination of age, BMI, alcoholism, and comorbidities were reported as factors likely to influence recurrence [14]. MSL may result in symptoms of a blockage of the upper airway. The preferred course of therapy for these individuals is tracheostomy combined with surgical lipectomy [15]. Just a few occurrences of orbital participation in MSL have ever been documented. Proptosis and persistent bilateral lumps on the eyelids may be seen in patients. It is possible to diagnose excessive bilateral non-capsulated fat deposits in the orbital fat, lower eyelids, salivary glands, subcutaneous tissue along the neck and behind the sternocleidomastoid muscles, and supraclavicular regions using CT scanning [16]. MSL may present with different symptoms. The classifications by Enzi et al. [17] and Donhauser et al. [18] are the old classifications. According to the new classification [19], type 1 is divided into three types: a, b, and c. Our patient had neck, shoulder girdle, upper arms, chest, abdomen, and upper and lower back involvement. The pseudoathletic appearance, which was type 2 according to the old classification, corresponds to type 1C in our patient according to the current classification. The very low female prevalence of this rare disease causes misdiagnosis in female patients, especially with obesity and systemic diseases. The familial mitochondrial MTTK c.8344A>G variant was found to be associated with MSL disease in all female family members [20]. Therefore, it is important to question the family history.

## Conclusions

In the type 1 presentation of MSL, the diagnosis is relatively easy because of the fat accumulation in the neck. However, the pseudo-athletic appearance of type 1C combined with the female patient can be confusing. Obesity and Cushing's syndromes may be considered first in the differential diagnosis. Diffuse non-capsulated fat accumulation on radiologic imaging and questioning of family history will lead to the correct diagnosis. In conclusion, multiple symmetric lipomatoses should be considered in the differential diagnosis of diffuse fat accumulation in the upper body in females.
